# Biochemical analysis reveals aberrant and variable Immunoglobulin M composition in Waldenström macroglobulinemia and IgM monoclonal gammopathy of unknown significance

**DOI:** 10.3389/fimmu.2025.1670408

**Published:** 2025-11-06

**Authors:** Nienke Oskam, Wouter Verhaar, Pleuni Ooijevaar-de Heer, Karima Amaador, Ninotska I. L. Derksen, Sofie Keijzer, Marie José Kersten, Marij Streutker, Josephine M. I. Vos, Theo Rispens

**Affiliations:** 1 Sanquin Research and Landsteiner Laboratory, Academic Medical Center, Amsterdam, Netherlands; 2 Department of Hematology, Amsterdam University Medical Center, University of Amsterdam, Cancer Center Amsterdam and Lymphoma and Myeloma Center Amsterdam, Amsterdam, Netherlands; 3 Amsterdam Institute for Infection and Immunity, Amsterdam, Netherlands; 4 Amsterdam University Medical Center (UMC), Vrije Universiteit Amsterdam, Molecular Cell Biology and Immunology, Amsterdam, Netherlands

**Keywords:** Waldenström macroglobulinemia, MGUS, antibodies, polymerization, paraprotein

## Abstract

Waldenström Macroglobulinemia (WM) is a rare B cell malignancy defined by greater than 10% infiltration of lymphoplasmacytic cells in the bone marrow (BM) and a circulating monoclonal Immunoglobulin M (IgM), while its precursor state IgM monoclonal gammopathy of undetermined significance (MGUS) has <10% BM infiltration. WM and IgM MGUS are unique amongst malignant lymphomas because symptoms and treatment indication may be caused by monoclonal IgM and not by the malignant cell infiltration. These symptoms correlate poorly with IgM levels, suggesting there may be specific biochemical properties of those pathological IgMs, yet IgM structure in IgM gammopathies has not been systematically studied. In healthy individuals, IgM circulates as a pentameric molecule that consists of five covalently linked monomers (H2L2 pairs), a joining (J-) chain and one CD5-Like (CD5L) molecule. In order to gain insight into structural variation of IgM in monoclonal IgM gammopathies, we developed and tested several assays to determine J-chain and CD5L content and polymerization state of IgM from 29 IgM MGUS and WM patients. In multiple cases, IgM was found to be (partially) devoid of J-chain, which associated with differential assembly of IgM into variably sized polymers. Moreover, we found that IgM exceeding ~5 g/L was no longer saturated with CD5L. Relative binding of polymeric Ig receptor varied by over 30-fold. Combined, in this pilot study we demonstrate that structural and functional variation in IgM of IgM MGUS and WM is common. These aberrations in IgM structure may relate to variations in clinical phenotype in IgM monoclonal gammopathies.

## 1 Introduction

Waldenström macroglobulinemia (WM) is a rare lymphoproliferative disorder, defined by the presence of a lymphoplasmacytic cell (LPL) infiltrate in the bone marrow (BM, ≥10% infiltration) and a circulating monoclonal immunoglobulin M (IgM). Compared to other lymphoproliferative disorders, clinical manifestations are highly heterogeneous in WM as they are not only related to marrow disease burden, but also to symptoms that are mediated by the monoclonal IgM. Symptoms attributed to monoclonal IgM include, but are not limited to, hyperviscosity syndrome, auto-immune complications such anti-MAG neuropathy and cold agglutinin disease, and deposition disorders such as IgM-related amyloid light-chain (AL) amyloidosis (1–5). From a clinical point of view, discordances between development of symptoms and IgM levels and/or tumor burden are common. Notably, even in the pre-malignant condition IgM monoclonal gammopathy of undetermined significance (MGUS), which by definition has an LPL infiltration below 10%, a significant proportion of patients may develop symptoms and even require treatment for complications related to the monoclonal IgM itself (2, 6). This condition has been termed monoclonal gammopathy of clinical significance (MGCS) (7).

Nowadays, total IgM levels and the monoclonal IgM fraction are assessed in routine diagnostics by means of nephelometry and electrophoresis, respectively (8). While higher serum (monoclonal) IgM levels are associated with risk of progression for both IgM MGUS and asymptomatic WM to symptomatic WM (5), they alone are insufficient predictors of the clinical phenotype (9, 10). Total IgM levels have only been shown to be somewhat predictive for the development of hyperviscosity syndrome in patients with IgM levels exceeding 60 g/L, although data on this are conflicting (11, 12). It is therefore likely that besides the concentration of IgM, other – structural – characteristics may play a role in the development of certain clinical manifestations, particularly in the setting of IgM MGCS. This hypothesis is further supported by a recent study showing similar serum IgM levels and BM histological features between patients with various IgM MGCS (mainly peripheral neuropathy) and those with IgM MGUS (13).

IgM is normally secreted as a complexed, pentameric molecule that consists of five covalently linked monomers (H2L2 pairs), and a joining (J-) chain. In addition, in circulation, one CD5-like (CD5L) molecule is covalently attached to each IgM pentamer, whereas secreted IgM associates noncovalently with the secretory component (SC), derived from the polymeric Immunoglobulin Receptor (pIgR) (14). The biochemical IgM composition in IgM monoclonal gammopathies has not been systematically studied. Prior small studies in a small number of WM patients, described IgM without J-chain (15, 16), but also other aberrant structural features have been reported (17–19). The absence of J-chain causes IgM to assemble as hexamers (and also smaller oligomers) (15, 16), which are known to be more efficient activators of the complement system compared with regular pentamers (20, 21). In addition, other *in vitro* studies demonstrate that IgM without J-chain is unable to incorporate CD5L (14), and shows an altered binding profile to known IgM receptors (22). Thus, variations in the IgM structure may have functional implications as well.

Taken together, these data suggest that there may be a significant role for specific biochemical properties of IgM in the pathogenesis of WM and/or MGCS, highlighting the need for systematic analysis of (monoclonal) IgM. Such studies remain however technically challenging, both because of IgM’s innate biochemical properties (i.e. its size and propensity for agglutination), as well as the lack of IgM-specific tools. We developed and tested several immunochemical assays to determine J-chain contents and polymerization status, as well as association with CD5L and the interaction with pIgR. Here, we present a first systematic exploration into the structural variation of IgM in twenty-nine patients with IgM MGUS or WM.

## 2 Methods

### 2.1 Serum samples

To gain insight into the possible compositional variation (i.e. J-chain integration and CD5L contents) among IgM in monoclonal IgM gammopathy, we selected samples of 29 patients diagnosed with IgM MGUS or WM (**Supplementary Table 1**), with selection of samples primarily based on varying levels of IgM. The definitions of IgM MGUS and WM were based on the WHO classification of 2017 (23). Samples were collected and processed by the B cell malignancy biobank at the Amsterdam University Medical Center (Amsterdam UMC). Written consent was received from all study participants. Serum from twenty tetanus boosted donors was obtained anonymously from leftover materials after routine diagnostics and taken along as healthy controls. These were used anonymously without any connection to person-specific data. The research was conducted in accordance with the Declaration of Helsinki.

### 2.2 IgM enzyme-linked immunosorbent assays

Several aspects of IgM composition were assessed by ELISA. For all assays, mouse-anti-human IgM (2 µg/mL, MH-15-1, Sanquin) was coated on maxisorp plates (Thermo Fisher Scientific) overnight at 4°C. Samples were warmed to 37°C prior to dilution. To determine IgM levels, samples were diluted in high performance ELISA (HPE) buffer (Sanquin). Coated plates were washed five times with phosphate-buffered saline (PBS) supplemented with 0.02% Tween-20 (PBS-T). Diluted samples (100 µl) were transferred to the plates and incubated for one hour at room temperature while shaking (300 rpm) and thereafter washed with PBS-T. Bound IgM was detected with horse radish peroxidase-labeled a-IgM (0.33 µg/mL, MH-15-1-HRP, Sanquin) in HPE and to determine IgM-bound CD5L, a-CD5L-HRP (0.5 µg/mL, clone 5B5) (14) in HPE was used. Both were incubated for 30min. Binding of the pIgR was assessed by incubation with biotinylated, recombinant pIgR (1 µg/mL, extracellular domains) (14) in PBS supplemented with 0.2% w/v gelatin (Merck) and 0.1% v/v Tween-20 for one hour followed by streptavidin-poly-HRP (100 ng/mL; Sanquin) for 30min.

In order to determine the fraction of IgM-J-chain, samples were pre-incubated under (very) mildly reducing conditions to selectively dissociate bound CD5L which interferes with the detection of the J-chain. Samples were diluted in Tris-buffered saline (TBS) supplemented with 1 mM glutathione (GSH, Sigma), 10 mM Ethylenediaminetetraacetic acid (EDTA) and 0.1% Tween-20 and incubated overnight at 37°C while shaking at 300 rpm. This condition allowed selective reduction of the disulfide bond connecting CD5L to IgM, while leaving the remainder of the IgM molecule intact (14). In case of fractionated healthy donor sera using HP-SEC (see below), 4.4 mg/mL human serum albumin (Albuman; Sanquin) was added to the dissociation buffer to compensate for low protein content. Samples were further diluted in TBS supplemented with 0.1% Tween-20 (TBS-T). Plates were washed five times with PBS-T and 100 µL sample was transferred to the plates. These were incubated at RT for an hour while shaking at 300 rpm and subsequently washed. IgM-J-chain was detected with an HRP-conjugated in-house-developed anti-J-chain (0.25 µg/mL, clone 1G5) in TBST.

Lastly, for all assays detection was visualized and the absorbance was read at 450 nm and 540 nm for background correction. Total IgM levels measured by ELISA were used for all healthy donors to determine J-chain content and IgM-bound CD5L. Given discrepant results of the IgM ELISA when levels exceed 50 g/L (**Supplementary Table 2**), nephelometry data was used to compare all other assays.

### 2.3 High-performance size-exclusion chromatography of serum samples

Sera from two healthy donors and four WM patients were diluted 1:1 and 1:4 respectively in PBS and then filtered using a 0.22-μm filter (Merck Millipore). The sera were then fractionated by HP-SEC by applying 200 µL of this dilution to a Superose 6 increase column (10/300 GL, GE Healthcare) and eluting it with PBS (0.5 mL/min) using the AKTA Go system (Cytiva). Fractions of 250 µL were collected, which were subsequently measured in ELISA and stored at -30°C.

### 2.4 IgM and J-chain Western blot

For western blot, serum samples were run on 4-12% Bis-Tris protein (NuPAGE, Invitrogen) or 3-8% Tris-Acetate (NuPAGE, Invitrogen) gels under reducing and non-reducing conditions respectively according to the manufacturer’s protocols. Approximately 1 µg of IgM was loaded per sample based on the levels determined by nephelometry. Bis-Tris gels were run at 200V for 55 minutes and Tris-Acetate gels were run at 150V for 2–3 hours, after which the proteins were transferred to a nitrocellulose membrane (iBlot transfer system, Thermo Fisher). IgM HC was detected with mouse-anti-human IgM (MH-15-1, Sanquin) and J-chain with mouse-anti-J-chain (MCA693, Bio-Rad) followed by goat anti-mouse IgG-HRP (GM-17-HRP, Sanquin). The proteins were visualized with Pierce ECL Western blotting Substrate (Thermo Fisher Scientific).

### 2.5 Statistical analysis and visualization

Graphs were made and statistical analysis was performed using Graphpad Prism v10.

## 3 Results

In this study, a total of twenty-nine sera of IgM MGUS and WM patients were included with IgM levels ranging from 1.1 to 58.2 g/L. Patient characteristics can be found in **Supplementary Table 1**. Because IgM composition is completely homogeneous and stable in the healthy population, no further information was gathered on the healthy controls.

### 3.1 J-chain-negative IgM and the presence of varying IgM polymers

**Figure 1:**
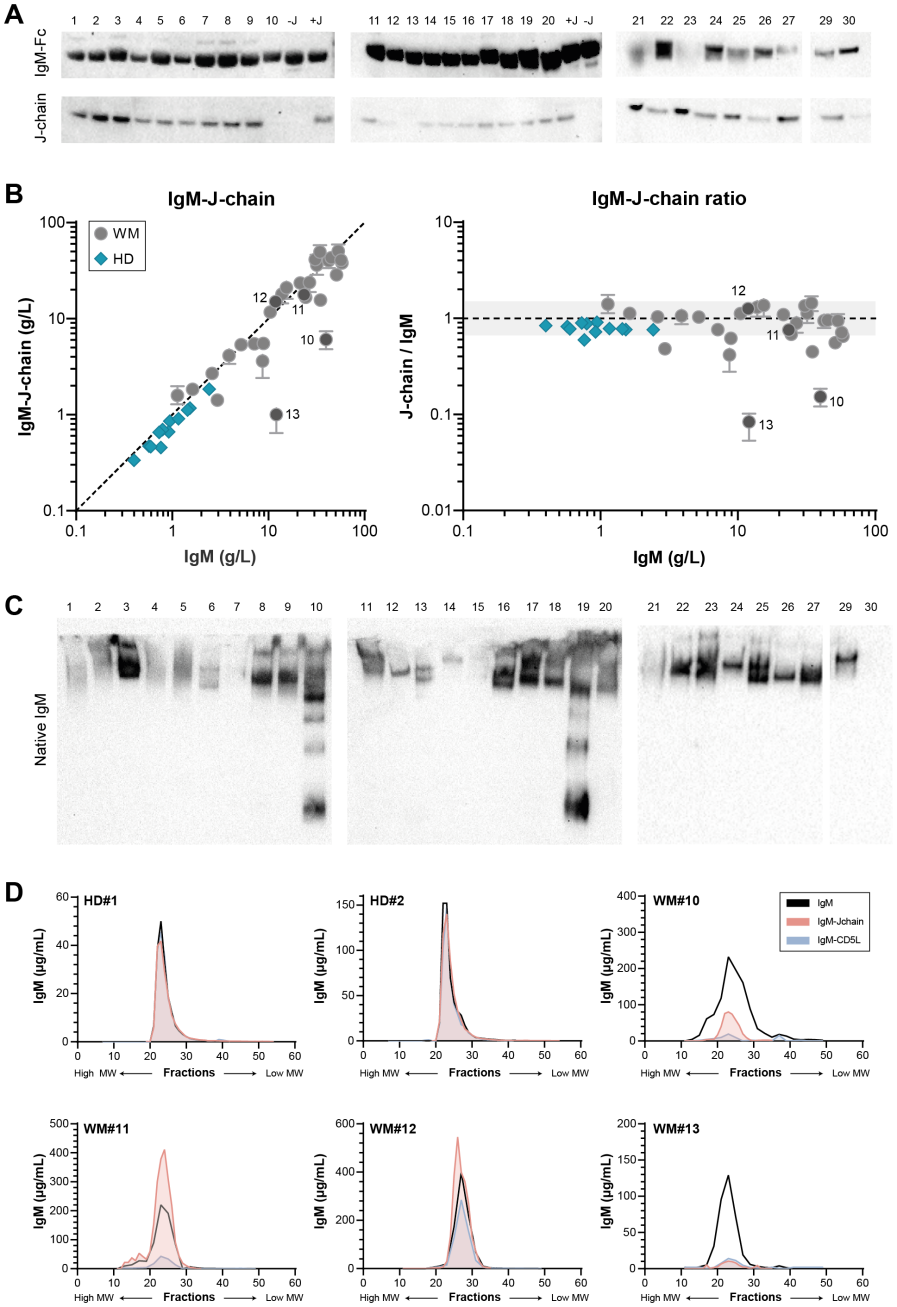
Variable J-chain contents and polymerization of IgM in patients with IgM MGUS and WM. **(A)** Western blot for IgM-Fc and J-chain of reduced SDS-PAGE (4-12% Bis-Tris) gel. IgM-Fc at ~75 kDa and J-chain at ~25 kDa. Sample 28 was later identified as an IgG-producing LPL and excluded from all analyses. **(B)** J-chain contents of serum IgM were assessed in IgM-J-chain ELISA for IgM MGUS and WM patients (n = 29) and healthy donors (HDs; n = 12). Data shown as median ± IQR of n = 3. The x- and y-axes are both presented on a log10 scale. **(C)** Western blot for IgM-Fc of native PAGE (3-8% Tris-acetate) gel. Bands show natively folded IgM polymers, with the largest polymers at the top of the gel and smaller polymers or monomers running lower. The IgM of sample 30 does not enter the native gel (**Supplementary Figure 1**) and therefore does not show a band. **(D)** Sera of two HDs and four WM patients (#10-13) were fractionated by size exclusion, after which IgM, IgM-J-chain and IgM-CD5L were measured by ELISA in each fraction.

We first assessed the presence of the IgM-Fc (constant region) and J-chains in each patient sample by western blot (**Figure 1A**), as J-chain-negative, hexameric IgM in WM patients has been reported in the past (15, 16). Detection of the J-chain shows that in some cases, IgM appears to be largely negative for J-chain (sample 10 and 13), whereas for others the J-chain content appears to be relatively low compared to the overall IgM level. To obtain a more precise quantification of J-chain content, we next measured the IgM-J-chain in an ELISA with a newly in-house developed anti-J-chain antibody and compared J-chain contents with total IgM levels assessed by nephelometry. IgM in healthy donors was made up of ~100% J-chain-containing IgM, whereas we found substantial variation within IgM of patients. IgM was at least partly J-chain deficient in many patients, and in a minority, IgM was even largely devoid of J-chain (again samples 10 and 13, in line with western blot results) (**Figure 1B**).

The presence of a J-chain dictates polymerization of IgM into the (regular) pentameric structure. Having demonstrated that the IgM molecule in a large fraction of patient samples is partly J-chain deficient, we next investigated the IgM polymerization state. IgM from each patient sample was visualized on native western blot (**Figure 1C**). Multiple bands can be appreciated for several samples, indicating the presence of IgM polymers of different sizes. A whole range of IgM polymers can be clearly detected for sample 10 and 19 especially, which contain relatively little J-chain overall.

To further zoom in on the relation between overall J-chain contents and the polymerization state of IgM, we fractionated four patient sera (#10-13) using size exclusion and subsequently determined the IgM and IgM-J-chain levels in the obtained fractions (**Figure 1D**). Multiple peaks representing IgM can be appreciated for all samples but sample #12, showing again the presence of different oligomers. The J-chain-containing peak corresponds to the pentameric fraction of IgM, whereas all other peaks are clearly devoid of J-chain. Molecules of larger size likely correspond to hexamers, whereas the other peaks are made up of smaller oligomers. In patients with only a fraction of IgM containing J-chain, the pentameric peak is expected to consist mainly of J-chain-negative pentamers (such as in WM13). Taken together, these assays show that in a subset of WM patients, IgM is (partly) devoid of J-chain, which leads to the assembly into different IgM polymers.

### 3.2 High levels of IgM are not saturated by CD5L

**Figure 2:**
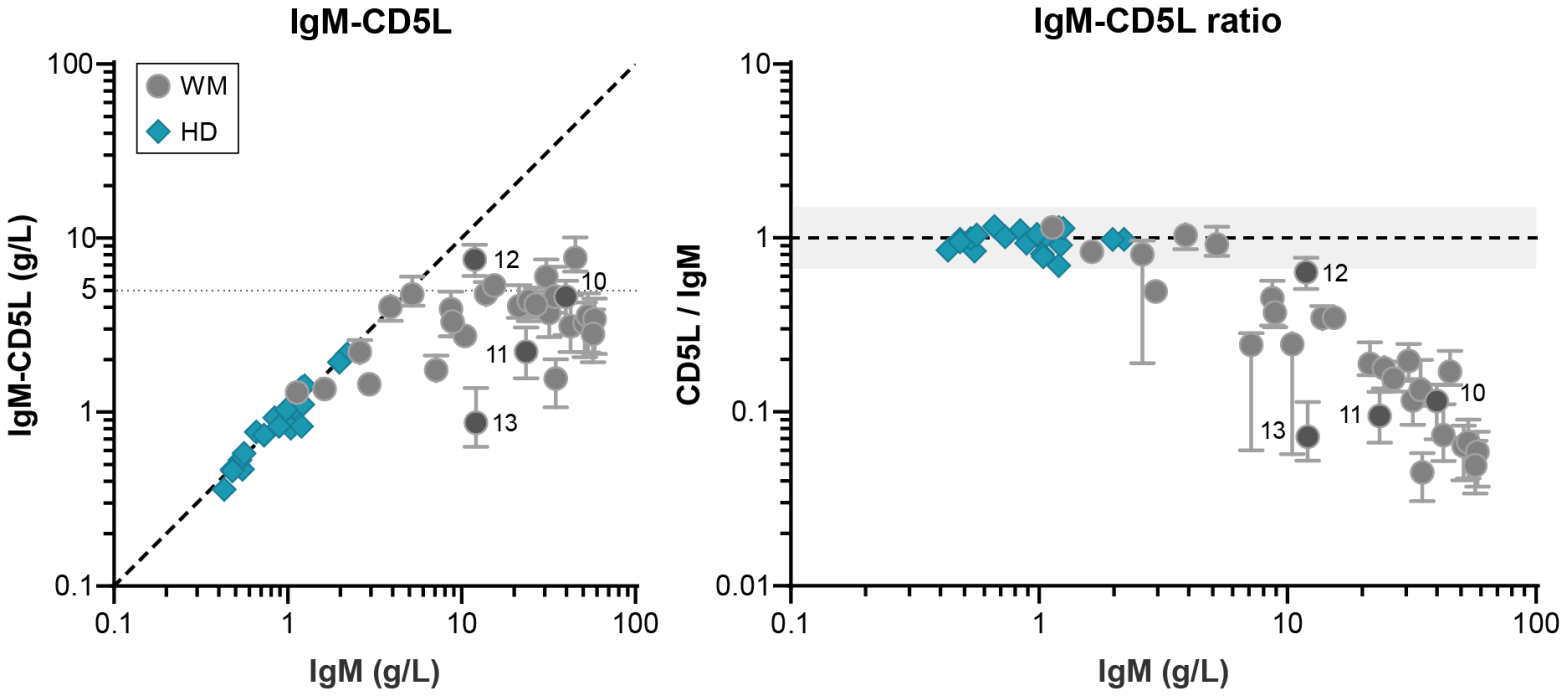
IgM-bound CD5L in patients with IgM MGUS and WM. Levels of IgM-CD5L were determined by ELISA for IgM MGUS and WM patients (n = 29) and HDs (n = 20) and compared to total IgM levels (as determined by nephelometer and ELISA respectively). As shown previously, the ratio between IgM and IgM-CD5L for HDs is ~1, showing that all IgM is in complex with CD5L, whereas this ratio is much lower for patients (~0.1; right panel). Data shown as median ± IQR of n = 3. The x- and y-axes are both presented on a log10 scale.

CD5L is a small 36 kDa protein that is associated with circulating IgM. One molecule of CD5L can bind IgM covalently in the gap that is created when a J-chain is integrated. In healthy individuals, all serum IgM is saturated with CD5L (14). However, the presence of J-chain is required for CD5L integration into IgM and since many of the IgM monoclonals are at least partly J-chain-deficient, we also determined the levels of IgM-CD5L (**Figure 2**). Whereas overall IgM levels and IgM-CD5L complexes correlate perfectly for donor sera, we find that IgM is saturated with CD5L in only a fraction of patients, in particular the ones with relatively low levels of IgM. The presence of CD5L-devoid IgM cannot be fully explained by the lack of J-chain in these samples. For example, IgM from patient 11 forms predominantly J-chain-containing pentamers, but only ~10% of IgM molecules contains CD5L (**Figure 1D**). The levels of IgM-CD5L complexes appear to plateau at approximately 5 g/L of IgM and all IgM exceeding that concentration is not associated with CD5L. This is further illustrated by the ratio between IgM-CD5L complexes and total IgM, which is ~1 for donors but ~0.1 (median) for patients with IgM MGUS and WM.

### 3.3 Differences in IgM composition lead to variable pIgR binding

**Figure 3:**
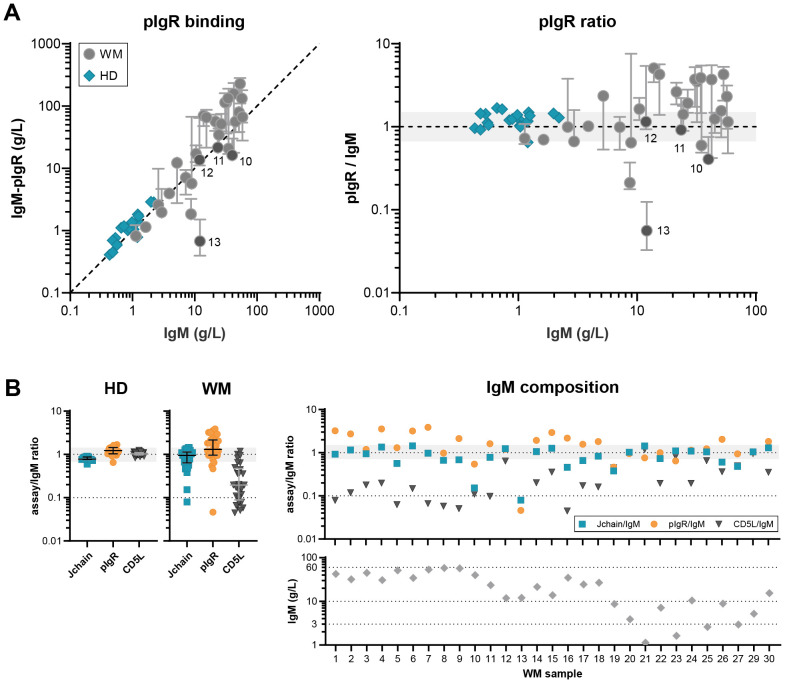
Variable IgM composition differently affects receptor binding. **(A)** pIgR binding to IgM was assessed in ELISA for IgM MGUS and WM patients (n = 29) and HD (n = 20) and compared to total IgM levels. Whereas HD IgM show the expected 1:1 interaction with pIgR, interaction with patient IgM is more variable. Data shown as median ± IQR of n = 3. The x- and y-axes are both presented on a log10 scale. **(B)** The ratio between the levels of total IgM and IgM-J-chain, pIgR and IgM-CD5L.

Lastly, we assessed the interaction of IgM and the pIgR in donor and patient samples (**Figure 3A**). This receptor binds directly to the J-chain of IgM (and also IgA) (24), binding that is slightly hindered by CD5L integration (14) and is therefore very dependent on IgM’s exact composition. Compared to donor IgM, binding to the pIgR shows a lot of variation in IgM of patients, with binding both better and worse than the reference serum. Absence of a J-chain completely abolishes binding which can be appreciated for patient 13 (**Figure 3B**), whereas absence of CD5L increases binding which can be seen for example in patient 1.

Overall, the fraction of IgM-CD5L is significantly lower for patients (p<0.001, t test assuming unequal variances, Welch’s correction), but J-chain content and pIgR binding capacity is not. However, by comparing the variances of the (log-transformed) ratio’s using an F test, we confirmed the hypothesis that the J-chain content, CD5L content, and pIgR binding capacity is much more variable for patients than HD (p<0.001). These results show that the exact and variable composition of IgM greatly impacts the way it interacts with one of its receptors.

## 4 Discussion

In this study, we investigated the biochemical composition, polymerization state and pIgR binding capacity of IgM in IgM MGUS and WM patients. Based on previous reports describing some cases of aberrant IgM structure (15–19), we systematically studied IgM from twenty-nine IgM MGUS and WM patients with a wide range of IgM levels, and show great, previously unknown, structural and functional heterogeneity.

IgM is normally assembled as a J-chain-containing, pentameric molecule that is complexed with one CD5L molecule. Other circulating compositions are not commonly found in healthy individuals (14). It is therefore highly surprising that aberrant IgM molecules, i.e. devoid of J-chain and/or CD5L, were found with such high frequency in approximately one third of IgM gammopathy patients in our study. This may simply be due to the extraordinary high levels of secreted IgM (in the case of CD5L), but may likely also be caused by yet undiscovered transcriptional alterations that occur during the development of LPL cells (in the case of J-chain). It is currently unclear what causes these LPL cells to secrete aberrant IgM without J-chain. J-chain is normally expressed during B cell development towards antibody-secreting cells (25). However, somatic mutations in the LPL cell may negatively impact the expression or production of J-chain, or otherwise impact the assembly of IgM.

As the J-chain dictates polymerization of IgM (26), it is likely that the high concentration of IgM and the partial lack of J-chain in these patients leads to a mixture of regular and aberrant IgM molecules. J-chain-negative, hexameric IgM has been shown to be a much more potent complement activator than regular IgM (20) and could therefore be of importance in the context of complement mediated disorders such as cold agglutinin disease (CAD), where monoclonal IgM leads to complement mediated hemolysis and erythrocyte agglutination (27–29). Additionally, hexameric IgM may also be involved in other auto-immune phenomena related to monoclonal IgM, such as anti-MAG peripheral neuropathy that has been related to complement activation (30). Furthermore, we hypothesize that these hexameric molecules may increase IgM’s propensity to develop hyperviscosity syndrome, as they have an altered three-dimensional structure, which is a known modulator for viscosity. In support of this, multiple retrospective studies (11, 12, 31) have not been able to specify a distinct IgM threshold above which patients developed hyperviscosity syndrome and required plasmapheresis.

On top of these possible contributions to IgM related symptoms, IgM without J-chain may also play a vital role in dysregulating immune response(s) in patients with IgM gammopathies. As is illustrated by binding to the pIgR in **Figure 3**, an altered receptor binding profile may be expected for many of the aberrant IgM forms. Moreover, the lack of CD5L, which is generally described as an anti-inflammatory molecule (32), may imply a more pathogenic fraction of IgM that next to, or in combination with, possible J-chain-negativity, could in turn contribute to the recently coined inflammatory WM (33, 34) and might thus influence the phenotype.

Taken together, these aberrant IgM variants, associated with potentially abnormal interactions of (monoclonal) IgM with IgM receptors and complement may explain the wide variation in clinical manifestations observed in IgM gammopathies. Given the current sample size and exploratory nature of the current study, this remains to be investigated in larger patient groups. Indeed, these data may pave the way to a new field of research in IgM gammopathies. This may include further exploration of the possible interplay of clinical phenotype with IgM composition by applying these techniques in larger cohorts with specific clinical phenotypes (e.g. MGCS, such as CAD and anti-MAG neuropathy), hyperviscosity syndrome, as well as longitudinally during transitions across different disease states. Furthermore, this may ultimately trigger development of treatments that target the pathological IgM component specifically.

In conclusion, we developed specific tools to assess IgM composition in IgM monoclonal gammopathies and demonstrated that there exists great structural and functional variation among patients with IgM MGUS and WM, while this variation was not seen in healthy donor IgM. Future studies could provide valuable insights into the link between heterogeneity in clinical manifestation and IgM composition.

## Data Availability

The raw data supporting the conclusions of this article will be made available by the authors, without undue reservation.
