# Supplementary material for: Biochemical analysis reveals aberrant and variable Immunoglobulin M composition in Waldenström macroglobulinemia and IgM monoclonal gammopathy of unknown significance
Source: Front Immunol. 2025 Nov 6;16:1670408. doi: 10.3389/fimmu.2025.1670408 (PMC12631112; doi:10.3389/fimmu.2025.1670408)
Supplement: Supplementary file 1 [file DataSheet1.docx]

Supplementary Material

**Supplementary Table 1** – Patient characteristics at baseline.

|  | Patients with IgM MGUS/WM (n=29) |
| --- | --- |
| Age (years), median (IQR) | 68 (13.5) |
| Male, n (%) | 18 (62) |
|  |  |
| Serum IgM (g/L), median (IQR) | 21.44 (29.2) |
| Serum IgG (g/L), median (IQR) | 6.2 (4.1) |
| Serum IgA (g/L), median (IQR) | 0.54 (0.64) |
| Hb (mmol/L), median (IQR) | 8.05 (1.6) |
|  |  |
| IgM MGUS, n (%) | 3 (10.3) |
| Asymptomatic/smoldering WM, n (%) | 9 (31.0) |
| Symptomatic WM, n (%) | 1 (3.4) |
| (Previously) treated WM patients, n (%) | 16 (55.2) |
| Stable Disease, n (%) | 3 (21.4) |
| Partial Response, n (%) | 7 (50.0) |
| Very Good Partial Response, n (%) | 4 (29.6) |
| Progressive disease, n (%) | 2 (6.9) |
|  |  |
| MYD88 L265P, n (%) | 23 (75.9) |
| Unknown, n (%) | 4 (13.8) |

**Supplementary Table 2** – IgM quantifications in IgM MGUS and WM sera by various methods. Nephelometry (Neph), Immunofixation Electrophoresis (IFE), Enzyme-linked immunosorbent assay (ELISA).

|  | Neph. | IFE | ELISA |
| --- | --- | --- | --- |
| # | IgM | M-protein | IgM |
| 1 | 42,2 | 27,5 | 410,5 |
| 2 | 31,94 | 21,4 | 98,9 |
| 3 | 45,07 | 26,6 | 92,1 |
| 4 | 30,69 | 21,1 | 134,5 |
| 5 | 51,18 | 28,1 | 109,1 |
| 6 | 34,34 | 21,5 | 81,5 |
| 7 | 53,34 | 32,3 | 188,5 |
| 8 | 58,2 | 37,3 | 96,1 |
| 9 | 57,02 | 24,4 | 103,6 |
| 10 | 39,82 | 18,4 | 204,5 |
| 11 | 23,51 | 12,1 | 24,7 |
| 12 | 11,89 | 6 | 25,5 |
| 13 | 12,07 | N/A | 17,5 |
| 14 | 21,44 | 12,3 | 39,4 |
| 15 | 13,83 | 6,8 | 38,6 |
| 16 | 23.51 | N/A | 29,4 |
| 17 | 24,44 | 16,1 | 27,6 |
| 18 | 26,73 | 18,8 | 83,8 |
| 19 | 8,69 | 2,9 | 6,2 |
| 20 | 3,89 | N/A | 6,4 |
| 21 | 1,13 | 0,4 | 1,9 |
| 22 | 7,14 | 2,4 | 9,5 |
| 23 | 1,63 | 0,3 | 2,1 |
| 24 | 10,45 | 5,4 | 15,7 |
| 25 | 2,6 | 1,1 | 5,3 |
| 26 | 8,89 | N/A | 14 |
| 27 | 2,94 | N/A | 3,6 |
| 29 | 5,19 | 2 | 8,4 |
| 30 | 15,4 | 10,5 | 37,8 |


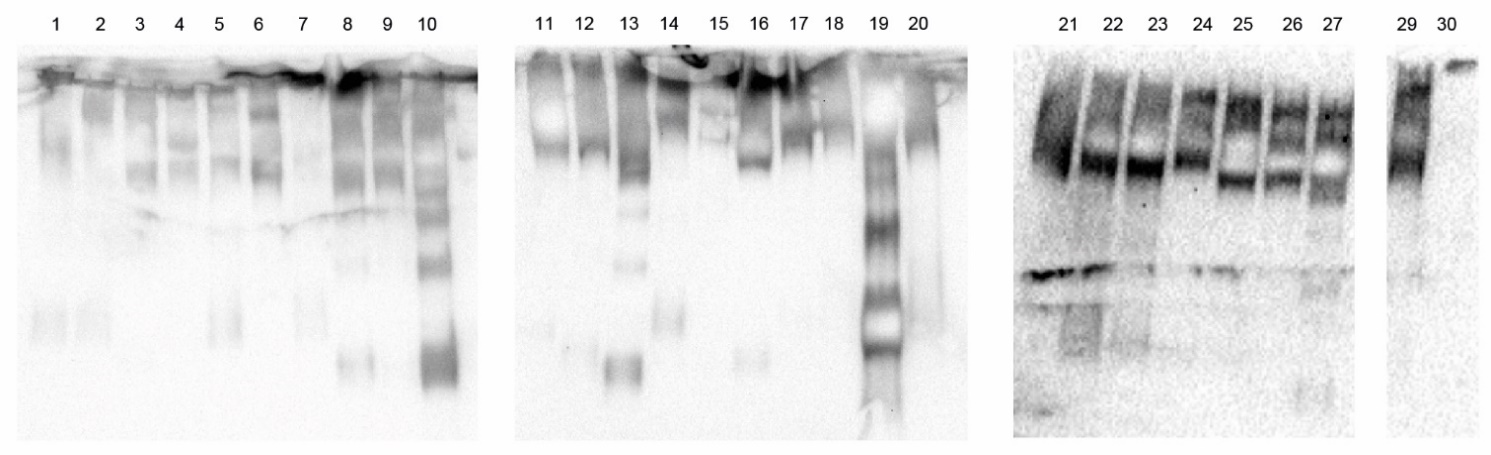


**Supplementary Figure 1 –** Smaller IgM species in IgM MGUS and WM sera. Western blot for IgM-Fc of native PAGE (3-8% Tris-acetate) gel. Bands show natively folded IgM polymers, with the largest polymers at the top of the gel and smaller polymers or monomers running lower. A higher concentration of sample was run to better visualize smaller bands.
